# Molecular Markers of Radiation Induced Attenuation in Intrahepatic *Plasmodium falciparum* Parasites

**DOI:** 10.1371/journal.pone.0166814

**Published:** 2016-12-02

**Authors:** Miranda S. Oakley, Nitin Verma, Hong Zheng, Vivek Anantharaman, Kazuyo Takeda, Yamei Gao, Timothy G. Myers, Phuong Thao Pham, Babita Mahajan, Nirbhay Kumar, Davison Sangweme, Abhai K. Tripathi, Godfree Mlambo, L. Aravind, Sanjai Kumar

**Affiliations:** 1 Division of Bacterial, Parasitic, and Allergenic Products, Center for Biologics Evaluation and Research, Food and Drug Administration, Silver Spring, Maryland, United States; 2 Division of Emerging and Transfusion Transmitted Diseases, Center for Biologics Evaluation and Research, Food and Drug Administration, Silver Spring, Maryland, United States; 3 National Center for Biotechnology Information, National Library of Medicine, NIH, Bethesda, Maryland, United States; 4 Division of Viral Products, Center for Biologics Evaluation and Research, Food and Drug Administration, Silver Spring, Maryland, United States; 5 Genomics Technologies Section, Research Technologies Branch, National Institute of Allergy and Infectious Diseases, NIH, Bethesda, Maryland, United States; 6 Bloomberg School of Public Health, Johns Hopkins University, Baltimore, Maryland, United States; INSERM, FRANCE

## Abstract

Experimental immunization with radiation attenuated sporozoites (RAS) and genetically attenuated sporozoites has proved to be a promising approach for malaria vaccine development. However, parasite biomarkers of growth attenuation and enhanced immune protection in response to radiation remain poorly understood. Here, we report on the effect of an attenuating dose of γ-irradiation (15 krad) on the *Plasmodium falciparum* sporozoite (PfSPZ) ultrastructure by electron microscopy, growth rate of liver stage *P*. *falciparum* in liver cell cultures, and genome-wide transcriptional profile of liver stage parasites by microarray. We find that γ-irradiation treated PfSPZ retained a normal cellular structure except that they were vacuous with a partially disrupted plasma membrane and inner membrane complex. A similar infection rate was observed by γ-irradiation-treated and untreated PfSPZ in human HCO-4 liver cells (0.47% versus 0.49%, respectively) on day 3 post-infection. In the microarray studies, cumulatively, 180 liver stage parasite genes were significantly transcriptionally altered on day 3 and/or 6 post-infection. Among the transcriptionally altered biomarkers, we identified a signature of seven candidate parasite genes that associated with functionally diverse pathways that may regulate radiation induced cell cycle arrest of the parasite within the hepatocyte. A repertoire of 14 genes associated with protein translation is transcriptionally overexpressed within the parasite by radiation. Additionally, 37 genes encode proteins expressed on the cell surface or exported into the host cell, 4 encode membrane associated transporters, and 10 encode proteins related to misfolding and stress-related protein processing. These results have significantly increased the repertoire of novel targets for 1) biomarkers of safety to define proper attenuation, 2) generating genetically attenuated parasite vaccine candidates, and 3) subunit candidate vaccines against liver stage malaria.

## Introduction

In 1967, immune protection induced by radiation attenuated sporozoites (RAS) vaccination was first documented in mice by intravenous administration of attenuated *P*. *berghei* sporozoites [1]. In 1973, immunization of man against RAS vaccination was demonstrated [2]. In 2002, Hoffman *et al* established that RAS vaccination of human subjects with approximately 1000 bites from irradiated *P*. *falciparum* infected mosquitoes provides essentially complete protection against sporozoite challenge [3]. In recent years, it was shown that cryopreserved RAS delivered by the intravenous [4] but not intradermal [5] route induced sterile protection in humans. While radiation-induced attenuation relies on genome-wide alterations, efforts are also underway for generation and clinical testing of genetically attenuated candidate malaria sporozoite vaccines by targeted deletion(s) in known molecules [6] [7].

It is well established that a dose of 15,000 rad (15 krad) is essential for both complete attenuation of the parasite and induction of sterile immunity [3]. At this attenuating dose, the sporozoite is capable of invading the liver cell but fails to mature into infectious liver stage merozoites. However, in two independent reports, Clyde *et al* [2] and Rieckman *et al* [8] have shown that immunization with sporozoites irradiated at 12 krad caused blood form infections in 6 of 18 immunized volunteers suggesting that the window of irradiation-induced attenuation is quite narrow. Attenuation at higher doses (20–27 krad) rendered the sporozoites ineffective as vaccinated volunteers developed malaria after challenge by infectious sporozoite bites [9]. These data strongly suggest that radiation, when delivered in an optimal dose, has both attenuating and immunogenic effects on malaria sporozoites. However, the parasite genes responsible for radiation-induced attenuation and induction of immune protection have not been studied.

In spite of significant investments in research towards antigen discovery (genomics and proteomics-based) and pre-clinical development, the majority of clinical trials against pre-erythrocytic stage malaria are still based on the circumsporozoite protein (CSP) of the sporozoite. However, none of the CSP-based vaccines have matched the effectiveness of the RAS-based approach which is thought to be mostly directed against the liver stage parasites. Therefore, to develop superior next generation candidate vaccines, it is imperative that the parasite antigens expressed following infection of human liver cells with RAS are identified and evaluated in clinical studies.

In this study, we determined the parasite molecular changes that regulate growth attenuation and enhanced immune protection induced by radiation. To accomplish this, we examined the effect of radiation on i) the ultrastructure of the sporozoite by electron microscopy, ii) the development of day 3 and day 6 *P*. *falciparum* liver stage parasites in human HCO-4 liver cells by immunofluorescence microscopy and iii) the liver stage parasite transcriptome on days 3 and 6 post PfSPZ infection by microarray. After extensive bioinformatic analyses, we identified a signature of liver stage *P*. *falciparum* parasite molecules that might contribute to both growth attenuation and increased immunogenicity induced by γ-irradiation. We propose that a subset of these parasite molecules could be evaluated as vaccine candidates against the *P*. *falciparum* liver stage parasite and may serve as gene targets to create the next generation of genetically attenuated sporozoite vaccines.

## Materials and Methods

### Preparation of *P*. *falciparum* Sporozoites

PfSPZ used for transmission electron microcopy, the liver stage parasite growth assay, and transcriptional profiling of intrahepatic parasites were prepared using the protocols described earlier [10] [11]. Three-5 days old female *An*. *Stephensi* mosquitoes were membrane fed with *P*. *falciparum* (NF54) gametocytes, cultured using human erythrocytes and serum. Cages containing gametocyte fed mosquitoes were maintained at 26°C, 80% relative humidity. Eight-9 days after feeding, 8 mosquitoes from each cage were taken out and their guts were dissected in order to count oocysts. On Day 15 after the gametocyte feed, mosquitoes from all cages were pooled in a single cage and then distributed in equal numbers to two cages. One cage was subjected to 15 krad radiation exposure from a ^137^Cs source of a Cesium irradiator and the second cage did not receive any radiation exposure. Next, irradiated and non-irradiated mosquitoes were transferred to a -20°C freezer for 20 minutes. When the mosquitoes were found to be inactivated completely, they were dipped in 70% Ethanol and transferred to a Petri-dish having MEM medium containing 5% human serum and 1x penicillin-streptomycin. Salivary glands of the mosquitoes were dissected and kept in 200 μl MEM medium containing 5% human serum and 1x penicillin-streptomycin (complete MEM medium) in an Eppendorf tube. Dissected glands were passed several times through a 26G needle fitted to a 1 ml plastic syringe in order to triturate the glands and release the sporozoites and then further purified onto a discontinuous Renografin-60 gradient.

### Transmission Electron Microscopy

Irradiation-treated (15 krad) and non-treated PfSPZ were used in transmission electron microscopy studies. Briefly, PfSPZ were fixed in 2% gluteraldehyde in PBS pH 7.3 overnight at room temperature and then rinsed in 0.1 M PBS buffer thrice overnight at 4°C. Samples were post-fixed for 1 hour with 1% osmium tetroxide, dehydrated at room temperature and rinsed with distilled water thrice at room temperature. Samples were stained with uranyl acetate in 50% ethanol for 15 minutes at room temperature, dehydrated in a series of ethanol buffers 50%, 70%, 95% and 100% ethanol thrice for 15 minutes and then 100% propylene oxide twice at room temperature. Samples were embedded in block molds in pure Epon 12 for 36 hours at 60°C. Thin sections were cut by ultra-microtome (Leica UC7) and stained with uranyl acetate and lead citrate and examined with a Zeiss EM 120 transmission electron microscope. Images of irradiated and control parasites were taken with Gaton 1000 XP camera system and scored blindly and analyzed to assess the structural changes in response to γ-irradiation.

### *P*. *falciparum* Liver Stage Parasite Growth Assay

Briefly, 40,000 HCO-4 cells were seeded in each of the eight wells of Lab-Tek II CC2 glass chamber slides one day before infection. 40,000 *P*. *falciparum* sporozoites (PfSPZ) either irradiated at 15 krad or non-irradiated, purified from the same batch of mosquitoes, in 50 μl DMEM/F-12 medium containing 10% FBS and 1x Penicillin-Streptomycin were added to each of the 8 wells of the respective slides containing HCO-4 cells. Slides were centrifuged for one minute at 110 x *g* prior to incubation for 3 hours at 37°C, 5% CO_2_, after which the PfSPZ suspension was aspirated off from each well and washed three times with complete DMEM/F-12 medium. PfSPZ infected HCO-4 cells were maintained at 37°C and 5% CO2 either for 3 days or 6 days with a daily change of medium.

### Immunofluorescence Microscopy

To determine the development and growth of liver stage parasites within HCO-4 cells upon infection with irradiated or non-irradiated PfSPZ, infected cells at day 3 and 6 post-infection were fixed with chilled methanol for 20 minutes at room temperature. The fixed cells were washed for five minutes each at room temperature on a rocker platform with three changes of 1X PBS, followed by blocking with 5% normal goat serum (Invitrogen) for one hour at 37°C. 100 μl of polyclonal rabbit anti-LSA1 antibody and anti-*P*. *falciparum* HSP70 mouse antibody (mAb 4C9) in PBS with 2% normal goat serum (NGS) and 0.001% saponin was added to each well of the LabTek slide and the slides were incubated at 37°C for 90 minutes. After three washing, five minutes each at room temperature, 100 μl of Alexa Fluor 488 goat anti-rabbit IgG (Invitrogen) or Alexa Fluor 488 goat anti-mouse IgG and 1:1000 dilution (2mg/ml stock) of DAPI (4',6-diamidino-2-phenylindole) in 0.02% Evans Blue, was added to each well of the LabTek slide. After one hour incubation at 37°C, wells of the slide were washed three times with PBS and slides were mounted with a coverslip using Fluoromount mounting medium (Electron Microscopy Sciences, Hatfield, PA). Slides were observed under a fluorescent microscope using 400x magnification with filters for green (Alexa Fluor 488), red (Evans blue) and blue (DAPI) colors. Images were captured from the same field and superimposed to get the final multicolored images.

### Preparation of RNA, Amplified cRNA Synthesis and Microarray Studies

Microarray was performed to measure the effect of γ-irradiation on the transcriptional profile of intrahepatic parasites after sporozoite invasion of HCO-4 human liver cells. Analysis of the parasite transcriptome by microarray was performed in quadruplicate comparing two biological replicates of RNA isolated from human liver cell cultures infected with untreated versus γ-irradiation treated sporozoites. Importantly, comparisons were made on both days 3 and 6 post sporozoite invasion of human liver cells. To perform microarray analysis of the parasite transcriptome, whole HCO-4 cell cultures containing intrahepatic parasites were harvested on days 3 and 6 post-infection of γ-irradiated treated verses untreated *P*. *falciparum* sporozoites, snap frozen in TRIzol^®^ (Life Technologies^™^, Carlsbad, CA), and stored at -80°C until use. For preparation of RNA, thawed samples were first homogenized with chloroform and RNA was then precipitated from the aqueous phase with isopropanol, washed with 75% ethanol, and resuspended in water for use in microarray experiments. RNA was then amplified and labeled with cy3 (untreated) or cy5 (γ-irradiated treated) dyes using the Low Input QuickAmp Labeling Kit (Agilent Technologies, Santa Clara, CA). Briefly, cDNA was synthesized from 200 ng of RNA template at 40°C for 2 hours in a reaction mixture containing AffinityScript-Reverse Transcriptase, Oligo dT-Promoter Primer, DTT, and dNTPs. Next, amplified labeled cRNA was generated from cDNA at 40°C for 2 hours in a reaction mixture containing T7 RNA polymerase, DTT, NTPs, and either cy3 (untreated) or cy5 (γ-irradiated treated) label. After amplification and incorporation of label, labeled cRNA transcripts were washed twice to remove unincorporated label and resuspended in RNase-free water using the RNeasy^®^ Mini Kit (Qiagen, Valencia, CA). Equivalent amounts of labeled cRNA transcripts were next hybridized to a custom-designed Agilent SurePrint oligonucleotide array (Agilent Technologies) with 44,000 features (including 5,254 distinct 60-mer probes for Pf 3D7 transcripts, each spotted 8 times). The microarray chip was then scanned at 5 micron resolution and image-analyzed using Feature Extraction software version 9 and the data was filtered using NIAID microarray database tools (mAdb.niaid.nih.gov) as previously described [12] [13] [14].

### Computational Analysis of Protein Sequences

Iterative sequence profile searches were run using the PSI-BLAST program against the non-redundant (NR) protein database of National Center for Biotechnology Information (NCBI) [15] [16]. Similarity-based clustering for both classification and culling of nearly identical sequences was performed using the BLASTCLUST program (ftp://ftp.ncbi.nih.gov/blast/documents/blastclust.html). The HHpred program was used for profile-profile comparisons [17]. Multiple sequence alignments were built by MUSCLE and KALIGN PCMA programs [18–21]. For previously known domains, the Pfam database was used as a guide, though the profiles were often augmented by addition of newly detected divergent members that were not detected by the original models. Signal peptides and transmembrane segments were detected using the TMHMM and Phobius programs [22, 23]. Phylogenetic analysis was conducted using an approximately-maximum-likelihood method implemented in the FastTree 2.1 program under default parameters [24, 25]. The in-house TASS package, a collection of PERL scripts, was used to automate aspects of large-scale analysis of sequences, structures and genome context.

## Results

### Effect of γ-Irradiation on the Ultrastructure of *P*. *falciparum* Sporozoites

**Fig 1:**
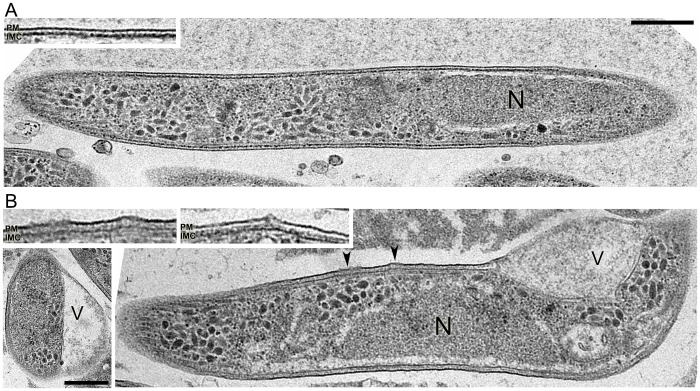
Thin-section electron microscopy of non-irradiated (top) and γ-irradiated at 15 krad (bottom) *Plasmodium falciparum* sporozoites (PfSPZ). γ-irradiated PfSPZ were wider in diameter and edematous and about 20% (20/100) have abnormal vacuous (V). Electron density of the inner membrane complex (IMC) of γ-irradiation treated PfSPZ were lower than non-treated PfSPZ. IMC near the vacuous shows much lower electron density or has disappeared. Plasma membrane (PM) of γ-irradiated PfSPZ was also partially disrupted (arrowheads) and the space between PM and IMC was wider in the irradiated group. Insets show higher magnification view of these changes. Center inset shows a γ-irradiated PfSPZ that had partial PM and IMC disruption. Bottom left panel shows short axis view of different irradiated sporozoites that had a large vacuous (V). There is no significant change of nuclei (N), endoplasmic reticulum, mitochondria, or apical complex structure seen in both control and irradiated groups. Bars indicate 500 nanometers.

We wanted to know the effect of exposure to an attenuating dose of γ-irradiation on the ultrastructure of PfSPZ. *Plasmodium* sporozoites are small elongated cells less than 1 μm in width and usually contain a single nucleus and a well-defined plasma membrane (PM) and inner membrane complex (IMC); the PM and IMC are separated by a narrow space and the cytoplasmic face of the IMC has been reported to contain electron dense material [26]. Fig 1 shows thin-section electron microscopy of untreated PfSPZ and irradiation treated PfSPZ (15 krad). Compared to untreated, irradiation-treated PfSPZ were wider in diameter and had an edematous appearance. In addition, we find that 20% (20/100) of γ-irradiation treated PfSPZ had a large abnormal vacuous as seen on the short axis view of PfSPZ (Fig 1). The electron density of the IMC in irradiation treated SPZ was lower compared to untreated. Importantly, in treated parasites, the IMC near the vacuous had a much lower electron density or had fully disappeared, their PM were partially disrupted or in some cases ruptured, and the space between the PM and IMC was wider. A higher magnification view further illustrated partial PM and IMC disruption (Fig 1, center inset). There were also minor but noticeable structural alterations in the nuclei, endoplasmic reticulum, mitochondria, or apical complex structure in PfSPZ that can be ascribed to γ-irradiation.

### Effect of γ-Irradiation on the Development of Liver Stage *P*. *falciparum* Parasites

**Fig 2:**
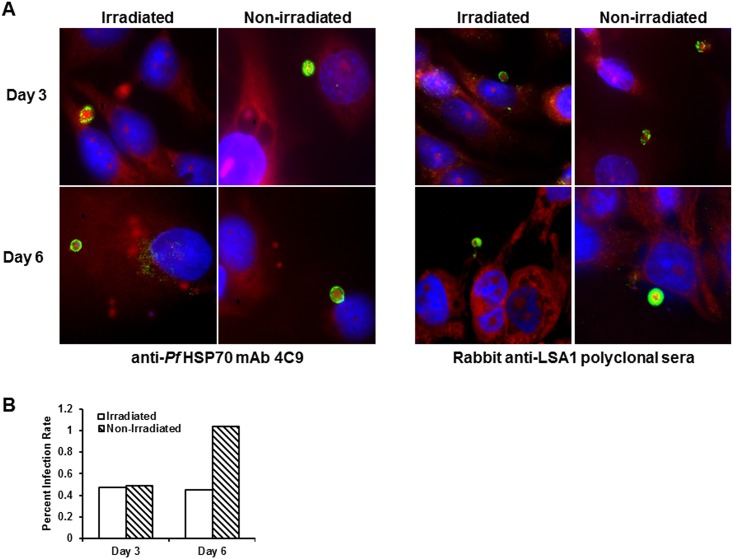
Fluorescence microscopy of human HCO-4 cells infected with γ-irradiated treated and untreated sporozoites. (A) HCO-4 cells were infected with sporozoites in a 1:1 ratio and parasites were then labeled with anti-HSP70 or anti-LSA-1 antibody on days 3 and 6 post-infection. (B) The percent infection rate (infected/total x 100) of HCO-4 cells was determined on day 3 and 6 post-infection of γ-irradiated treated verses untreated sporozoites by enumeration of 1000 HCO-4 cells stained with anti-HSP70.

We next determined if exposure of PfSPZ to an attenuating dose of γ-irradiation (15 krad) influenced their ability to invade and develop inside human HCO-4 cells as measured on day 3 and day 6 post-PfSPZ infection. Intrahepatic parasites were reacted with anti-HSP70 antibody and anti-LSA1 antibody in IFA and the rate of infection and progression from day 3 to day 6 hepatic stage parasites were determined by microscopy (Fig 2). Results showed that on day 3, γ-irradiation treated (0.47%) and untreated (0.49%) PfSPZ had a similar rate of infection in HCO-4 cells, as revealed by staining with anti-HSP70 antibody, indicating that γ-irradiation had no apparent effect on the infection rate and early liver stage development. In the γ-irradiation treatment group, an infection rate of 0.45% was observed on day 6 suggesting that liver form parasites were still visible during the late stages of the developmental cycle (Fig 2).

### γ-Irradiation-Induced Alterations in the Transcriptome of Liver Stage *P*. *falciparum*

To determine the molecular mechanisms of attenuation of the intrahepatic parasite induced by γ-irradiation, we performed microarray analysis in quadruplicate on RNA isolated from HCO-4 liver cells infected with non-irradiated versus γ-irradiated parasites on days 3 and 6 post-invasion. Whole infected liver cell cultures were used for RNA preparation. A gene was considered transcriptionally altered if it fulfilled three criteria based on fold change in expression and statistical significance: 1) was ≥2 fold upregulated or downregulated in three of the four (75%) microarray replicates 2) had a ≥ 2 fold increase or decrease in the average ratio of expression and 3) had a *p* value ≤ 0.05 (2-tailed Student *t* test). For the day 3 dataset, the input data was 5815 genes. 5607 genes were excluded by criteria 1 and 2 (fold change) and 71 genes were excluded by criterion 3 (statistical significance) resulting in 137 statistically significant genes (65.7% upregulated, 34.3% downregulated). To validate this transcriptome dataset, we performed an additional microarray on independent day 3 *P*. *falciparum* liver cell cultures and Boolean comparison demonstrated a 76.6% (105 of the 137 genes) overlap in the two datasets. This indicates that our microarray is robust in identifying transcriptionally altered genes induced by γ-irradiation. On day 6, the input data was 5816 genes. 5705 genes were excluded by criteria 1 and 2 (fold change) and 49 genes were excluded by criterion 3 (statistical significance) resulting in 62 statistically significant genes (93.5% upregulated, 6.5% downregulated). Among the day 3 and 6 datasets, a total of 180 genes were transcriptionally altered by γ-irradiation and 10.6% of the genes were differentially expressed on both day 3 and 6 post-infection. Of the 180 genes, 170 genes were identified as having domains that shed some light on their biochemical/biological functions. Based on these and previously published literature surveys, the genes were systematically categorized into functional classes (Table 1 and S1 Table).

**Table 1:** Biologic Functions of Proteins Encoded by *Plasmodium falciparum* Liver Stage Parasite Genes in Microarray Studies That Are Regulated by γ-Irradiation.

Protein, by Biological Function	Gene(s)	Day 3	Day 6
**Cell Cycle**			
G10 protein	PF3D7_0522800	2.3	
**Chromatin**			
histone acetyltransferase	PF3D7_0416400	8.3	
SET domain protein	PF3D7_0629700	6.7	
histone deacetylase	PF3D7_1472200	2.5	
**Cytoskeleton**			
probable protein, unknown function	PF3D7_1428400	6.4	6.6
conserved Plasmodium protein, unknown function	PF3D7_0529000	4.5	2.9
conserved Plasmodium protein, unknown function	PF3D7_0305200	3.6	
mitotic-spindle organizing protein 1	PF3D7_1339800	-2.7	
conserved Plasmodium protein, unknown function	PF3D7_1425500	-2.3	
tubulin—tyrosine ligase	PF3D7_1009700	-2.3	
**DNA binding**			
conserved Plasmodium protein, unknown function	PF3D7_1442700	-3.5	
**DNA repair**			
DNA mismatch repair protein MSH2	PF3D7_1427500		4.6
conserved protein, unknown function	PF3D7_1202100.1 PF3D7_1202100.2	6.4	
DNA-3-methyladenine glycosylase	PF3D7_1467100	-2.1	
**DNA repair; RNA**			
conserved Plasmodium protein, unknown function	PF3D7_1333900		15.3
**DNA Replication**			
DNA replication licensing factor MCM6 (MCM6)	PF3D7_1355100	-2.6	
**Metabolism**			
glycine cleavage T protein	PF3D7_1365500	3.0	
riboflavin kinase / FAD synthase family protein	PF3D7_1359100	-2.5	
**Metabolism (Lipid)**			
lysophospholipase	PF3D7_1476800	2.6	
**Metabolism (Nucleotide)**			
aspartate carbamoyltransferase (ATCase)	PF3D7_1344800	10.4	
**Nuclear envelop**			
conserved Plasmodium protein, unknown function	PF3D7_1446500	-2.5	
**Protein folding (FeS assembly)**			
iron-sulfur assembly protein	PF3D7_0207200	5.5	
**Protein folding/Stress response**			
DnaJ protein	PF3D7_1126300	8.4	9.4
heat shock protein 101,chaperone protein ClpB2 (HSP101)	PF3D7_1116800	3.6	7.6
DnaJ protein	PF3D7_1401100	2.1	
exported protein family 1 (EPF1)	PF3D7_1101800	-2.4	
conserved Plasmodium protein, unknown function	PF3D7_1448700	-2.5	
conserved Plasmodium protein, unknown function	PF3D7_1037900	2.4	
**Protein processing**			
stromal-processing peptidase	PF3D7_1440200	-2.3	
serine repeat antigen 3 (SERA3)	PF3D7_0207800	6.3	
methionine aminopeptidase 1a	PF3D7_0527300	4.7	
**Protein processing/Autophagy**			
cysteine protease ATG4	PF3D7_1417300		2.4
**Protein processing; Surface/exported protein**			
rhomboid protease ROM3 (ROM3)	PF3D7_0828000	4.5	
**RNA**			
RAP protein	PF3D7_0526000		5.3
RAP protein	PF3D7_1470600		2.2
RAP protein	PF3D7_1453600	10.5	4.2
PfMNL-3 mitoNEET-like iron-sulfur protein	PF3D7_1022900		4.5
large subunit rRNA processing RRM protein	PF3D7_1020000		4.1
RNA-binding protein	PF3D7_1002400.1 PF3D7_1002400.2	14.2	4.5
RNA-binding protein	PF3D7_0606100	3.4	3.7
zinc finger protein	PF3D7_1358600	2.2	
ATP dependent DEAD-box helicase	PF3D7_0623700	-2.2	
Ran-binding protein	PF3D7_1353400	-2.6	
mRNA cleavage factor-like protein	PFA_0450c	-2.2	
**RNA metabolism**			
DEAD box helicase	PF3D7_1307300		5.9
cell cycle control protein	PF3D7_1238300	12.4	
exosome complex exonuclease RRP44 (DIS3)	PF3D7_1359300	6.2	
early transcribed membrane protein 14.2 (ETRAMP14.2)	PF3D7_1476100	2.3	7.3
conserved Plasmodium protein, unknown function	PF3D7_0930100	-2.1	
conserved Plasmodium protein, unknown function	PF3D7_0921100	-2.2	
conserved Plasmodium protein, unknown function	PF3D7_0412200	-2.8	
conserved Plasmodium protein, unknown function	PF3D7_1411500	-3.1	
**RNA metabolism; chromatin**			
DNA/RNA-binding protein Alba 3 (ALBA3)	PF3D7_1006200	6.7	
**RNA;chromatin**			
endonuclease/exonuclease/phosphatase family protein	PF3D7_0319200		4.1
**RNA;Ubiquitin**			
CCR4-NOT transcription complex subunit 4	PF3D7_1235300	3.2	
**Signaling**			
adenylate kinase (AK1)	PF3D7_1008900		2.9
serine/threonine protein kinase	PF3D7_1247500		2.4
serine/threonine protein kinase, FIKK family (FIKK9.3)	PF3D7_0902200		2.4
GPI ethanolamine phosphate transferase 3	PF3D7_1214100		2.4
kinase	PF3D7_0514800		2.3
GTP-binding protein	PF3D7_1459100	15.1	
conserved Plasmodium protein, unknown function	PF3D7_1332200	7.1	
protein kinase	PF3D7_0926300	5.8	
Rab GTPase activator and protein kinase	PF3D7_0724000	5.1	
ras GTPAse	PF3D7_0616700	2.0	
conserved Plasmodium protein, unknown function (PFA_0305c) mRNA, complete cds	PFA_0305c	-2.3	
calcium-binding protein	PF3D7_0605400	-2.3	
14-3-3 protein	PF3D7_1422900	-3.0	
atypical protein kinase, ABC-1 family	PF3D7_1414500	3.7	
serine/threonine protein kinase, FIKK family, pseudogene (FIKK14)	PF3D7_1476400	3.2	
**Surface/exported protein**			
Plasmodium exported protein, unknown function	PF3D7_0532600		6.1
conserved Plasmodium protein, unknown function	PF3D7_0721100		5.4
acyl-CoA-binding protein	PF3D7_1119000		5.3
palmitoyltransferase	PF3D7_1321400		5.1
conserved Plasmodium membrane protein, unknown function	PF3D7_1216100		4.6
conserved Plasmodium protein, unknown function	PF3D7_1229700		3.7
conserved Plasmodium membrane protein, unknown function	PF3D7_1462600		3.4
rifin,PIR protein (RIF)	PF3D7_1254800		2.6
conserved Plasmodium protein, unknown function	PF3D7_1225700		2.4
erythrocyte binding antigen-181 (eba-181) mRNA, complete cds	PFA_0125c		2.3
rifin,PIR protein (RIF)	PF3D7_1400500		2.3
hemolysin III (HlyIII)	PF3D7_1455400		2.1
Plasmodium exported protein, unknown function	PF3D7_1252500		2.1
conserved Plasmodium membrane protein, unknown function	PF3D7_0820600	12.0	
conserved Plasmodium membrane protein, unknown function	PF3D7_1142300	11.1	
rifin,PIR protein (RIF)	PF3D7_0937400	10.2	
conserved Plasmodium protein, unknown function	PF3D7_0918400	9.9	
phosphatidylserine synthase	PF3D7_1366800	8.8	
conserved Plasmodium membrane protein, unknown function	PF3D7_1324300	6.7	
antigen 332, DBL-like protein (Pf332)	PF3D7_1149000	5.0	
erythrocyte membrane protein 1 (PfEMP1), exon 2	PF3D7_0223300	4.3	4.8
Plasmodium exported protein, unknown function, pseudogene	PF3D7_1478700	3.4	5.8
rifin,PIR protein (RIF)	PF3D7_0400900	3.1	-2.5
sporozoite invasion-associated protein 1 (SIAP1)	PF3D7_0408600	3.1	
conserved Plasmodium membrane protein, unknown function	PF3D7_0721500	3.1	
rifin,PIR protein (RIF)	PF3D7_0223100	2.9	
Plasmodium exported protein (hyp9), unknown function	PF3D7_0220600	2.8	
N6-adenine-specific methylase	PF3D7_1350700	2.7	
stevor,PIR protein	PF3D7_1149900	2.6	
rifin,PIR protein (RIF)	PF3D7_0200600	2.6	
Plasmodium exported protein (PHISTb), unknown function	PF3D7_1201000	2.5	
rifin,PIR protein (RIF)	PF3D7_1300500	2.5	
Pfmc-2TM Maurer's cleft two transmembrane protein (MC-2TM)	PF3D7_1100800	2.5	
conserved Plasmodium protein, unknown function	PF3D7_0719100	2.3	
Plasmodium exported protein (hyp15), unknown function	PF3D7_0425200	2.1	
stevor, pseudogene,PIR protein, pseudogene	PF3D7_1100700	2.1	
conserved Plasmodium membrane protein, unknown function	PF3D7_1411100.1 PF3D7_1411100.2	-2.2	
rifin,PIR protein (RIF)	PF3D7_0413300	-2.2	
conserved Plasmodium protein, unknown function	PF3D7_1105800	-2.2	
conserved Plasmodium protein, unknown function	PF3D7_0318000	-2.3	
conserved Plasmodium protein, unknown function	PF3D7_0921500	-2.4	
conserved Plasmodium membrane protein, unknown function	PF3D7_0523700	-2.6	
conserved Plasmodium membrane protein, unknown function	PF3D7_1336300	-2.7	
conserved Plasmodium protein, unknown function	PF3D7_1002600	-3.0	
conserved Plasmodium protein, unknown function	PF3D7_1369600	-3.3	
6-cysteine protein (B9)	PF3D7_0317100	4.0	
**Trafficking**			
AP-4 complex subunit epsilon	PF3D7_0904100	-2.4	
**Trafficking(mitochondrial)**			
mitochondrial import inner membrane translocase subunit TIM13	PF3D7_1242900	2.4	
mitochondrial inner membrane TIM10 associated protein	PF3D7_0502900	-2.1	
**Trafficking(vesicular)**			
transmembrane emp24 domain-containing protein	PF3D7_0422100		6.2
CGI-141 protein homolog	PF3D7_0419200	3.2	
conserved Plasmodium protein, unknown function	PF3D7_1107200	-3.8	
ras-related protein RAB7 (RAB7)	PF3D7_0903200	3.8	
**Transcription**			
DNA-directed RNA polymerase 2 8.2 kDa polypeptide	PF3D7_0708100	4.3	
DNA-directed RNA polymerase III subunit C	PF3D7_1421400		2.2
conserved Plasmodium protein, unknown function	PF3D7_0312000	2.6	
conserved Plasmodium protein, unknown function	PF3D7_1458800		3.4
**Translation**			
translation initiation factor IF-1	PF3D7_1469000		4.3
eukaryotic translation initiation factor 3, subunit 6	PF3D7_0528200		2.8
tetQ family GTPase	PF3D7_1235400		2.5
40S ribosomal protein S11	PF3D7_0317600	23.3	
translation elongation factor EF-1, subunit alpha	PF3D7_1123400	21.4	6.7
translation initiation factor IF-2	PF3D7_0607000	14.3	
60S ribosomal protein L44 (RPL44)	PF3D7_0304400	13.1	4.1
60S ribosomal protein L24	PF3D7_1309100	6.8	
cysteine—tRNA ligase	PF3D7_1015200.1 PF3D7_1015200.2 PF3D7_1015200.3	4.8	3.1
nucleic acid binding protein	PF3D7_1132300	4.8	
peptide chain release factor	PF3D7_0704900	4.6	
mitochondrial ribosomal protein L28 precursor,	PF3D7_1456600	-2.8	
**Translation;RNA**			
rRNA processing and telomere maintaining methyltransferase	PF3D7_0925200	8.0	
**Transport**			
vacuolar proton translocating ATPase subunit A	PF3D7_0806800		3.6
conserved membrane protein, unknown function	PF3D7_0719500	11.4	5.3
ABC transporter, (CT family) (MRP2)	PF3D7_1229100	7.6	
CorA-like Mg2+ transporter protein,	PF3D7_1304200.2	6.0	
mitochondrial carrier protein	PFA_0415c	-2.5	
**Ubiquitin**			
sentrin-specific protease 1 (SENP1)	PF3D7_1233900	4.5	
conserved Plasmodium protein, unknown function	PF3D7_1442800		-2.3
ubiquitin fusion degradation protein 1 (UFD1)	PF3D7_0916500	14.8	
ubiquitin carboxyl-terminal hydrolase isozyme L3 (UCHL3)	PF3D7_1460400	4.0	
RING zinc finger protein	PF3D7_0209700	2.9	
ubiquitin (Ub)	PF3D7_0815700	-2.3	
ubiquitin carboxyl-terminal hydrolase	PF3D7_1414700	-3.1	
cullin-like protein	PF3D7_0629800	-2.4	
**Apicomplexia only**			
conserved Plasmodium protein, unknown function	PF3D7_1009900		6.0
conserved Plasmodium protein, unknown function	PF3D7_1407700		4.7
conserved Plasmodium protein, unknown function	PF3D7_1228400	9.0	8.7
**Plasmodium only**			
conserved Plasmodium protein, unknown function	PF3D7_0506400		2.1
conserved Plasmodium protein, unknown function	PF3D7_0517100		-2.1
conserved Plasmodium protein, unknown function	PF3D7_1031100		-2.4
conserved Plasmodium protein, unknown function	PF3D7_0708200	9.4	3.7
conserved Plasmodium protein, unknown function	PF3D7_0802900	-2.3	
conserved Plasmodium protein, unknown function	PF3D7_1465600	-2.3	
conserved Plasmodium protein, unknown function	PF3D7_1137600	-2.7	
conserved Plasmodium protein, unknown function	PF3D7_1413100	-3.2	
conserved Plasmodium protein, unknown function	PF3D7_1362800		3.7
conserved Plasmodium protein, unknown function	PF3D7_1018100	19	

### Biologically Relevant *P*. *falciparum* Liver Stage Genes Regulated by γ-Irradiation

To decipher a possible correlation between regulation of gene expression by γ-irradiation and growth attenuation or enhanced immune protection, we systematically categorized the transcriptionally altered genes by their function (Table 1). Some of the biological relevant genes and their associated pathways are discussed below.

#### Effect of γ-Irradiation on DNA Repair and Replication

*Pf*. DNA-3-methyladenine glycosylase (PF3D7_1467100), an enzyme that initiates base excision repair, decreases in expression by -2.1 fold on day 3 post-infection and *Pf*. DNA mismatch repair protein MSH2p (PF3D7_1427500) increases in expression by 4.6 fold on day 6 post-infection in response to γ-irradiation (Table 1). Furthermore, PF3D7_1202100.2, which encodes a protein that contains the recently identified autoproteolytic SRAP domain with a key role in nucleotide excision repair [27], was found to be 6.4 fold elevated in expression on day 3 post-infection. In addition, the *P*. *falciparum* ortholog of Sen1, the central helicase subunit coordinating DNA replication with transcription and preventing errors that occur when replication forks encounter transcribed regions, is 15.3 fold overexpressed on day 6. *Pf*. DNA replication licensing factor (MCM6) (PF3D7_1355100) is also transcriptionally altered by -2.6 fold on day 3 post-infection in γ-irradiated treated versus untreated sporozoites. The MCM6 protein is a subunit of the prereplication helicase complex and is critical for DNA replication. Therefore, it is conceivable that down-regulation of *Pf*. MCM6 contributes to the growth attenuation induced by γ-irradiation of sporozoites.

#### Effect of γ-Irradiation on Parasite Chromatin

Three histone-modifying enzymes were found to be upregulated upon γ-irradiation. Of these, two (PF3D7_0416400: a HAT1-like acetyltransferase and PF3D7_1472200: PfHda1; a histone deacetylase) catalyze opposite reactions suggesting that there is probably an editing of the histone acetylation marks upon irradiation. The third histone modifying enzyme, PF3D7_0629700 (PfSET1), is a SET domain histone methyltransferase with methylated histone binding PHD fingers and an acetylated histone binding bromodomain. This enzyme is a member of the Trithorax family of SET domains and is proposed to function as a H3K4 methylase, which generates chromatin marks that are conducive to the transcriptional activation of previously silent genes, including surface antigens.

PF3D7_1006200 (*Pf*. Alba3) is upregulated by 7.19 ± 1.74 fold on day 3 post-invasion of hepatocytes in response to irradiation. Previous studies in the intraerythrocytic development of the parasite have shown that *Pf*. Alba3 binds to telomeric, subtelomeric, and *var* gene promoter regions of the chromosome and the DNA binding affinity of *Pf*. Alba3 is increased by deacetylation by *Pf*. Sir2A [28]. Furthermore, *Pf*. Alba3 has also been shown to be a component of a multiprotein complex engaged in the storage of translationally silent mRNAs that is critical for zygote to ookinete transformation [29]. Similarly, it is a component of *Trypanosoma brucei* RNA granules associated with starvation stress and trypanosome differentiation [30] [31]. Specifically, Subota *et al* demonstrated that overexpression of Alba3 perturbs differentiation of *T*. *brucei* from the midgut to the proventricular stage in the tsetse fly. This result leads us to speculate that γ-irradiation induced upregulation of *Pf*. Alba3 may similarly impair differentiation of the attenuated liver stage parasite into blood form merozoites by acting either at the level of chromatin or by binding cytosolic mRNA.

#### Transcription and RNA-Processing

Four basal transcription factors were found to be upregulated in either the day 3 or 6 post-infection samples. One of these is the DNA-directed RNA polymerase III, subunit C (PF3D7_1421400), which is responsible for transcribing tRNA and 5S RNA molecules essential for translation [32], and is upregulated on day 6 in response to γ-irradiation.

In contrast, 15 genes related to RNA-processing, modification and binding were elevated in our dataset. Among these was *Pf*. CCR4 (PF3D7_0319200), a nuclease subunit of the CCR4-NOT complex, the primary cytoplasmic deadenylase that shortens mRNA poly (A) tails. Consistent with this result, PFA_0450c, a gene encoding a key subunit of the complex required for mRNA cleavage for polyadenylation, is down-regulated by -2.2 fold. This suggests that the polyadenylated mRNA fraction might be decreased in response to irradiation. Similarly, *Pf*. Dis3 (PF3D7_1359300) is upregulated by 6.28 ± 0.36 fold on day 3 post-infection of hepatocytes. This protein has both endonuclease (PIN) and 3'-5' exonuclease (RnaseII) domains and is part of the exosome complex involved in 3'-5' RNA processing and tRNA degradation. In yeast, Dis3 is a ribonuclease that is essential for mitotic control and has been shown to increase in abundance in response to DNA replication stress [33, 34] suggesting that the DNA damage caused by the ionizing irradiation might also induce *Pf*. Dis3 in this situation.

*P*. *falciparum* contains a lineage-specific expansion of 11 members of the RAP domain family [35]. Interestingly, three of these 11 members are overexpressed by the parasite in response to irradiation on day 6 post-infection of the hepatocyte. Using sequence profile methods, we were able to show that these RAP domain proteins are not RNA-binding domains as previously proposed [35] but endonucleases of the restriction-endonuclease fold. They are likely to cleave RNA based on evidence from their role in trans-splicing of mRNAs in *Chlamydomonas* [36]. Our detection of expression of these RAP proteins provides the first hint regarding the function of this expanded family in *P*. *falciparum*. We propose that they might be involved in specific RNA processing events associated with stress-responses, such as irradiation-induced damage.

#### Translation Apparatus

12 *P*. *falciparum* genes coding for proteins which are part of the translation apparatus are overexpressed in response to γ-irradiation (Table 1). On day 3, genes that encode four ribosomal proteins, *Pf*. translation initiation factor IF-2 (PF3D7_0607000), *Pf*. translation elongation factor EF-1 subunit alpha (PF3D7_1123400), *Pf*. cysteinyl tRNA synthetase, and *Pf*. peptide chain release factor (PF3D7_0704900), which is required for termination of translation, are upregulated. Moreover, expression of *Pf*. rRNA processing and telomere maintaining methyltransferase (PF3D7_0925200), the methyltransferase responsible for m1A 645 base modification of the 25S rRNA, is also elevated by 8.0 fold on day 3 post-infection. Furthermore, on day 6, genes that encode one ribosomal protein, two translation initiation factors, *Pf*. translation elongation factor EF-1 subunit alpha (PF3D7_1123400), *Pf*. cysteinyl tRNA synthetase (PF10_0149), *Pf*. tetQ family GTPase (PF3D7_1235400), and *Pf*. deoxyhypusine hydroxylase (PF3D7_1302600), which activates eukaryotic initiation factor 5A, are also upregulated by γ-irradiation.

#### The Ubiquitin System

The ubiquitin system may be defined as the system for the conjugation and removal of ubiquitin and ubiquitin-like proteins (e.g. SUMO). We found that four genes associated with the ubiquitin system of the parasite are upregulated, whereas five genes are downregulated in response to γ-irradiation. Of interest is *Pf*. cullin-like protein (PF3D7_0629800), which is down-regulated by -2.42 ± 0.28 fold on day 3 post-infection with γ-irradiation-treated versus untreated sporozoites. Given the role of homologous cullin-containing E3 ligase in anaphase progression, genome replication [37] [38], and in damaged rRNA decay [39], it is possible that the downregulation of *Pf*. cullin-like protein might have a role in the growth attenuation observed in the parasite.

*Pf*. sentrin specific protease 1 (SENP1), the thiol peptidase required for deSUMOylation and activation of SUMO precursors (SUMO is a small ubiquitin-related modifier) prior to conjugation [40], is upregulated by 5.36 ± 2.06 on day 3 post-infection by γ-irradiation. Experiments using small molecule inhibitors indicate that *Pf*.SENP1 is essential for growth during the intraerythrocytic stage of the parasite [41]. Studies in other eukaryotes suggest that SENP1 is required for stress-induced mitotic arrest [42] and apoptosis [43], pointing to a possible role for the overexpression of this enzyme in growth attenuation.

*Pf*. ubiquitin fusion degradation protein 1 (PF3D7_0916500) is strongly upregulated (14.8 fold) by γ-irradiation on day 3 post-infection. *Pf*. Ufd1 is the polyubiquitin binding substrate-recruiting subunit of the conserved Cdc48-Npl4-Ufd1 complex that facilitates the proteasomal degradation of ER membrane proteins and cytoplasmic proteins. Its presence in our dataset suggests that the response to irradiation might involve clearance of damaged proteins via the above complex.

#### Protein Folding and Processing

Six genes related to protein folding and processing are upregulated and three are downregulated in response to γ-irradiation (Table 1). *Pf*. rhomboid protease 3 (ROM3) is upregulated by 5.89 ± 2.95 fold on day 3 post-infection of hepatocytes in response to γ-irradiation treatment of sporozoites. Loss of function analysis of *P*. *berghei* ROM3 indicates that this protease is essential for sporogony [44]. *Pf*. serine repeat antigen 3 (SERA3) is also upregulated by 8.36 ± 2.89 fold on day 3 post-infection. *Pf*. SERA3 is a member of a family of nine putative cysteine proteases that are thought to play a role in egress of blood stage parasites from erythrocytes and sporozoites from oocysts [45]. Studies in murine *P*. *berghei* malaria have shown that *Pb*. SERA3 is highly expressed during late liver stage, is released into the hepatocyte cytoplasm, and may contribute to hepatocyte death.

In contrast, the expression of *Pf*. stromal-processing peptidase (PF14_0382) decreases by -2.37 ± 0.27 fold on day 3 post-infection in response to γ-irradiation. A study by van Dooren *et al* demonstrated that cleavage of the N-terminal transit peptide by *Pf*. stromal-processing peptidase is required for protein export to the apicoplast organelle of the parasite [46]. Its ortholog in *Arabidopsis* is needed for chloroplast function and its loss results in developmental arrest after the 16-cell stage [47]. Likewise, compromised apicoplast function due to downregulation of this enzyme might contribute to the growth defect of the parasite that occurs in response to γ-irradiation.

#### Membrane-Associated and Exported Proteins

Remarkably, one of the largest effects of γ-irradiation is seen in the form of the upregulation of parasite surface proteins, with at least 27 of them in the set of 180 genes identified in our study (Table 1). Interestingly, *Pf*. palmitoyltransferase (PF3D7_1321400) is up-regulated by 2.17 ± 0.23 fold on day 3 and 6.79 ± 2.55 on day 6 post-infection of hepatocytes. This protein is one of 12 *P*. *falciparum* palmitoyltransferases that catalyzes the post-translational addition of a 16 carbon lipid moiety onto cysteine residues via a thioester bond [48] and is a key modification that allows membrane translocation. Over 400 palmitoylated proteins have been identified in intraerythrocytic stage parasites [49] and modification of proteins by elevated PF3D7_1321400 protein activity could accentuate their membrane association. It is possible that this upregulation of a diverse set of parasite antigens might play an important factor for immune protection induced by vaccination with whole PfSPZ attenuated by γ-irradiation.

## Discussion

By electron microscopy, we find that irradiation-treated PfSPZ had maintained their overall structural integrity although a few changes were noted. These sporozoites were wider in diameter with an edematous appearance, had lower electron density in the IMC, partial PM and IMC disruption, and a large abnormal vacuous in some (20%) parasites (Fig 1). Nonetheless, these structural alterations did not inhibit the ability of irradiation treated PfSPZ to invade and progress into liver form parasites; infection in HCO-4 cells was similar in the γ-irradiation treated and untreated groups on day 3, and slightly higher in the untreated group on day 6 post-PfSPZ infection (Fig 2). While in this study, we used HCO-4 cells, a routinely used cell line for *in vitro* studies on the intrahepatic stage of *P*. *falciparum* [50] [5], improved development of liver form *P*. *falciparum* in cryopreserved primary human hepatocytes has recently been reported [51] [52] Based on or results, and other published reports, we infer that primary human hepatocytes may be a better system than the HCO-4 cell line to study the development and growth of liver form *P*. *falciparum* parasites [53].

Although a previous study has measured the effect of γ-irradiation on the expression of a small subset (ten) of genes in the sporozoite prior to liver cell invasion [54], the effect of γ-irradiation on the intrahepatic *P*. *falciparum* parasite transcriptome at the whole genome level has not been performed. We performed transcriptome analyses on day 3 and day 6 liver form parasites to identify the γ-irradiation induced parasite biomarkers associated with attenuation and sterilizing immunity. Using this approach, we identified 180 parasite genes that were significantly altered in expression by γ-irradiation and assigned each gene to a functional category (Table 1).

A major objective of this study was to identify the parasite genes that regulate γ-irradiation induced developmental arrest of the parasite in the hepatocyte. This information in turn can be used to predict novel genetic targets to define sufficient attenuation in whole sporozoite-based vaccines and create genetically attenuated parasite vaccine candidates by gene deletion. Studies in malaria have not yet defined the effect of the majority of *P*. *falciparum* genes included in our dataset on the regulation of the growth and development of the malaria parasite. Therefore, we systematically surveyed the literature to determine whether there was a correlation between the gene expression of *P*. *falciparum* orthologs and the rate of cell growth and differentiation in other organisms. We identified seven candidate genes that may contribute to γ-irradiation induced developmental arrest of the parasite.

Due to the low invasion rate and parasite/host RNA ratio in liver cells, genome-wide transcriptional profiling of intrahepatic parasites has not previously been successful. A salient feature of this study was addition of a RNA template amplification step increased the sensitivity of the microarray assay by increasing the quantity of parasite specific cRNA transcripts available for hybridization to *P*. *falciparum* oligonucleotide probes. On the other hand, qPCR lacked the sensitivity to detect parasite transcripts due to the low abundance of *P*. *falciparum* transcripts in total RNA isolated from infected HCO-4 cells.

Three of the seven signature genes (*Pf*. mitotic control protein dis3, *Pf*. Alba3, and *Pf*. sentrin specific protease 1) are upregulated and four (*Pf*. DNA-3-methyladenine glycosylase, *Pf*. minichromosome maintenance complex subunit 6, *Pf*. cullin-like protein, and *Pf*. stromal-processing peptidase) are downregulated in response to γ-irradiation.

*Pf*. dis3 (upregulated by 6.28 ± 0.36 fold on day 3 post-infection) encodes a ribonuclease that is essential for mitotic control of the fission yeast *Schizosaccharomyces pombe* [33, 34]. *Pf*. Alba3, a DNA binding protein, is upregulated by 7.19 ± 1.74 fold on day 3 post-infection of hepatocytes. Overexpression of Alba3 in *T*. *brucei*, the protozoan parasite that causes sleeping sickness, impairs differentiation of the trypanosome in the tsetse fly [31]. *Pf*. sentrin specific protease 1 (SENP1), a SUMO protease, is upregulated by 5.36 ± 2.06 fold on day 3 post-infection of hepatocytes. Avian and human SENP1 are essential for stress induced mitotic arrest [42] and apoptosis [43], respectively. Based on these findings in other organisms, we can hypothesize that γ-irradiation induced upregulation of *Pf*. mitotic control protein dis3, *Pf*. Alba3, and *Pf*. SENP1 may adversely affect the growth and development of the parasite in the hepatocyte.

*Pf*. DNA-3-methyladenine glycosylase, an enzyme that is essential for base excision repair, is downregulated by -2.28 ± 0.49 fold on day 3 post-infection of hepatocytes. In the *Arabidopsis thaliana* plant, expression of DNA-3-methyladenine glycosylase was elevated in rapidly dividing tissue and correlated with DNA replication [55]. Furthermore, the human orthologue N-methylpurine DNA glycosylase (MPG) has been shown to negatively regulate cell cycle arrest and is overexpressed in several types of cancers [56]. *Pf*. minichromosome maintenance complex subunit 6 (MCM6), a helicase that is essential for DNA replication, is downregulated by -2.61 ± 0.27 fold on day 3 post-infection of hepatocytes. *Pf*. cullin-like protein, a ubiquitin ligase, is downregulated by -2.42 ± 0.28 fold on day 3 post-infection of hepatocytes. Cell cycle studies with *Saccharomyces cerevisiae* have demonstrated that Cul8, an ortholog of *Pf*. cullin-like protein [37], is required for anaphase progression [38]. Lastly, *Pf*. stromal-processing peptidase, which is essential for export of parasite proteins to the apicoplast organelle, is downregulated by -2.37 ± 0.27 fold on day 3 post-infection of hepatocytes. Interestingly, knockout mutations in the stromal processing peptidase of the *Arabidopsis* plant cause developmental arrest after the 16-cell stage [47]. The results of these studies suggest that γ-irradiation induced down-regulation of *Pf*. DNA-3-methyladenine glycosylase, *Pf*. MCM6, *Pf*. cullin-like protein, and *Pf*. stromal-processing peptidase may contribute to developmental arrest of the parasite.

A second major contribution of this study was identification of transcriptional changes in intrahepatic attenuated parasites that may contribute to the enhanced immune protection induced by treatment of sporozoites with γ-irradiation. Previously, we measured the effect of febrile temperature [12] and γ-irradiation [57] on the transcriptome and growth of intraerythrocytic stage parasites. Treatment of intraerythrocytic parasites with elevated temperature and γ-irradiation adversely effected *P*. *falciparum* growth and resulted in a systemic down-regulation of the translational machinery of the parasite. However, our data suggests that in intrahepatic parasites, translation might be buttressed by the strong upregulation of several genes that might allow a robust response to the damage caused by γ-irradiation. It remains unclear as to why γ-irradiation has disparate effects on translation-related genes in blood versus liver stage parasites.

It is important to address whether the elevated expression of certain translation apparatus components by γ-irradiation of sporozoites enhances immune protection. Interestingly, *Plasmodium yoelii* L3 ribosomal protein was recently identified as an antigenic target of memory CTLs induced by immunization with RAS, genetically attenuated parasites, and wildtype sporozoites with chloroquine prophylaxis, indicating that ribosomal proteins can be important targets of malaria specific CD8^+^ T cells [58]. However, we hypothesize that irradiation induced upregulation of the translational machinery of the parasite may result in an increase in the pool of parasite proteins available for immune presentation either as processed epitopes on the hepatocyte surface or as released antigens from apoptotic infected hepatocytes and, is thereby a contributing factor for the sterilizing immunity observed by RAS vaccination. Indeed, at least 27 of the 180 altered genes in our dataset are parasite surface proteins that are upregulated. For example, serine repeat antigen 3, antigen 332, DBL-like protein, early transcribed membrane protein 14.2, and several members of the *rif* and *stevor* gene families are selectively upregulated at the transcriptional level by γ-irradiation. This together with the fact that 10.6% of genes in the dataset were differentially expressed on both days 3 and 6 raises the possibility of prospecting for vaccine candidates that can target parasites through the early developmental stages and developing liver-form merozoites. In summary, our studies have identified a gene signature of growth attenuation induced by γ-irradiation and novel vaccine candidates for evaluation against pre-erythrocytic stage malaria.

## Supporting Information

S1 Table Computational Analysis of the microarray dataset. (PDF)
