# Supplementary material for: Molecular Markers of Radiation Induced Attenuation in Intrahepatic Plasmodium falciparum Parasites
Source: PLoS One. 2016 Dec 2;11(12):e0166814. doi: 10.1371/journal.pone.0166814 (PMC5135057; doi:10.1371/journal.pone.0166814)
Supplement: S1 Table — (PDF) [file pone.0166814.s001.pdf]

Supplemental Table 1

GI	Day 3 av	Day 6 av	Final arch	function	notes	pfam	runprofiles	rps	Pfam	gene	fa	
1E+08	2.28		SIG+G10 (ZnR?)	Cell cycle		G10	SIG	-	G10	PFE1140c	147	G10 protein
3E+08	8.324		Histone Acetyltransferase (2 or 3 repeats)	Chromatin		Hat1_N+RNA_po	ACET+ACET+IMF	ACET+MIP-T3+A	Hat1_N+RNA_pol_3_	PFD0795w	1259	conserved, unknown function, Pf3D7
3E+08	6.747		PHD+PHD+PHD+BROMO+PHD+SET+LRRNT	Chromatin		Med3+Utp14+PH	PHD+PHD+BROM	MIP-T3+Termin	Med3+Utp14+PHD+	PFF1440w	6753	SET domain protein, putative Pf3D7
3E+08	2.485		TM+TM+TM+ANK+DEACET+DEACET (histo	Chromatin		TM+TM+TM+Ank	TM+TM+TM+AN	ANK+Tox-DDRG	Ank_5+Med3+Hist_	PF14_069	2251	histone deacetylase, putative Pf3D7
3E+08	6.381	6.568	BetaPropeller+BetaPropeller+BetaPropeller	Cytoskeleton		WD40+Herpes_L	BetaPropeller+B	BetaPropeller+	WD40+Herpes_LMP	PF14_026	1990	probable protein, unknown function Pf3D7
1E+08	4.483	2.894	SIG+BetaPropeller+BetaPropeller+TM	Cytoskeleton		SIG+VCBS+VCBS+	SIG+IG	BetaPropeller	VCBS+VCBS	PFE1445c	719	conserved, unknown function, Pf3D7
1E+08	3.563		Low comp (and fibrinogen-hhpred)	Cytoskeleton		SMC_N	-	sigma	SMC_N	PFC0235w	1139	conserved, unknown function, Pf3D7
1E+08	0.372		MOZART1	Cytoskeleton		MOZART1	-	-	MOZART1	PF13_021	72	conserved, unknown function, Pf3D7
1E+08	0.44		RIIa(Regulatory subunit of type II PKA-R)	Cytoskeleton	hhpred			-	-	PF14_023	175	conserved, unknown function, Pf3D7
3E+08	0.426		TTL-ATPgrasp+ATPgraspC (TTL6/13)	Cytoskeleton		TTL	TTL-ATPgrasp+A	TTL-ATPgrasp+A	TTL	PF10_009	553	tubulin-tyrosine ligase, putative Pf3D7
1E+08	0.289		ZnR	DNA binding	Plasmodium	-	-	TRANSGLUTAM	-	PF14_040	2511	conserved, unknown function, Pf3D7
1E+08	-	4.605	MutS_III+MutS_V	DNA repair		MutS_III+MutS_V	-	SbcC+SbcC	MutS_III+MutS_V	PF14_025	811	DNA mismatch repair protein Msh2p, Pf3D7
1E+08	6.364		SRAP/BB1717-like(DUF159,1zn6A)	DNA repair		DUF159+Utp14+I	SRAP+IMP+SRAF	SRAP+MIP-T3+S	DUF159+Utp14+DUF	PFL0105w	401	conserved, unknown function, Pf3D7
1E+08	0.469		Pur_DNA_glyco	DNA repair		Pur_DNA_glyco	-	-	Pur_DNA_glyco	PF14_063	501	DNA-3-methyladenine glycosylase, Pf3D7
1E+08	-	15.32	SF-I-Helicase	DNA repair;RNA		Viral_helicase1+	IES1+RNA-Helica	RNA-Helicase	Viral_helicase1+AAA	PF13_018	1328	conserved, unknown function, Pf3D7
1E+08	0.389		MCM-AAA	DNA replication		MCM_N+MCM	MCM-AAA	MCM-AAA	MCM_N+MCM	PF13_029	929	replication licensing factor, putatative Pf3D7
1E+08	2.959		GCV_T+GCV_T_C (Aminomethyltransferase	Metabolism		GCV_T+GCV_T_C		-	GCV_T+GCV_T_C	PF13_034	406	aminomethyltransferase, mitochondrial Pf3D7
1E+08	0.399		Flavokinase	Metabolism		Flavokinase	-	-	Flavokinase	MAL13P1.	707	riboflavin kinase / FAD synthase family, Pf3D7
1E+08	2.574		Abhydrolase_6	metabolism (Lipid)		Abhydrolase_6	7TMR-HDED+3H	-	Abhydrolase_6	PF14_073	371	lysophospholipase, putative Pf3D7
1E+08	10.37		SIG+OTCace_N+OTCace (Aspartate carbom	Metabolism (Nucleotide)		SIG+OTCace_N+C	SIG	-	OTCace_N+OTCace	MAL13P1.	375	aspartate carbamoyltransferase Pf3D7
3E+08	20.84	5.606	TM+TM+WD40	Misc		Mito_carr+Mito_	TM+TM+McrB-A	TRANSGLUTAM	Spore_YhcN_YlaJ+Sp	PFI1120c	4255	conserved, unknown function, Pf3D7
1E+08	-	6.047	Conserved DxD with Fast_1	Misc	Apicomplexa	Vfa1	-	CDC27	Vfa1	PF10_009	779	conserved, unknown function, Pf3D7
1E+08	-	4.691	PF14_0074-Conserved domain (PF14_0074	Misc	Apicomplexa	-	-	-	-	PF14_007	406	conserved, unknown function, Pf3D7
3E+08	-	2.133	SIG+Plasmodium-specific(PFE0315c aln)	Misc	Plasmodium	SIG	SIG	-	-	PFE0315c	116	conserved, unknown function, Pf3D7
3E+08	-	0.487	SIG+Lowcomplexity(Fast_1 LRR?)	Misc	Plasmodium	SIG	SIG	-	-	PFE0855c	476	conserved, unknown function, Pf3D7
3E+08	-	0.418	PF10_0304. Pfa only. Conserved Ds (PF10_	Misc	Plasmodium	-	FUNGAL-DEAM-I	-	-	PF10_030	184	conserved, unknown function, Pf3D7
1E+08	9.366	3.703	Lowcomp+TM+Lowcomp+C.H..H….C	Misc	Plasmodium	TM+DUF2076	TM	-	DUF2076	PF07_002	954	conserved, unknown function, Pf3D7
1E+08	8.964	8.683	Low comp+Sugar-Tranferase??(hhpred-PFL	Misc	Apicomplexa	Innate_immun+T	-	sigma+TRANSG	Innate_immun+TFIIF	PFL1375w	1401	conserved, unknown function, Pf3D7
1E+08	4.645		Lowcomp	Misc		IncA+IncA+TT_ORF1		MIP-T3+TRANS	IncA+IncA+TT_ORF1	PFE1485w	1906	conserved, unknown function, Pf3D7
1E+08	0.445		Lowcomplexity	Misc		SMC_N+SMC_N+DUF3300		sigma+EP1+sigr	SMC_N+SMC_N+DU	PFL0350c	2612	conserved, unknown function, Pf3D7
3E+08	0.441		Plasmodium only	Misc	Plasmodium	-	-	-	-	MAL8P1.1	135	conserved, unknown function, Pf3D7
3E+08	0.429		TM+TM+Plasmodium-specific	Misc	Plasmodium	TM+TM	TM+TM	-	BioY	PF14_062	485	conserved, unknown function, Pf3D7
1E+08	0.389		LowComp	Misc				-	-	PFI0640c	789	conserved, unknown function, Pf3D7
3E+08	0.369		Lowcomplexity	Misc	Plasmodium	YfhO+Med3	-	-	YfhO+Med3	PF11_038	656	conserved, unknown function, Pf3D7
1E+08	0.311		(PF14_0130-aln)	Misc	Plasmodium only			-	-	PF14_013	573	conserved, unknown function, Pf3D7
1E+08	0.393		Nucleoporin_FG-repeat	Nuclear envelop		Nucleoporin_FG+Nucleoporin_FG		SbcC+TRANSGL	Nucleoporin_FG+Nu	PF14_044	3001	conserved, unknown function, Pf3D7
1E+08	5.496		HesB-like Iron Sulfur scaffold domain	Protein folding (FeS asse	2d2a_A ; PF0	-	-	-	-	PFB0320c	160	iron-sulfur assembly protein, putative Pf3D7
1E+08	8.359	9.408	TPR+DNAJ	protein folding/Stress response		DnaJ	DNAJ	DNAJ	DnaJ	PF11_027	640	DNAJ protein, putative Pf3D7
1E+08	3.58	7.557	SIG+ClpABN-AAA+ClpABC-AAA	protein folding/Stress response		SIG+Clp_N+AAA+	SIG+ClpABN-AAA	SbcC+ClpABN-A	Clp_N+AAA+OmpH+	PF11_017	906	heat shock protein 101, putative Pf3D7
3E+08	2.068		DnaJ+DnaJ-X+DnaJ-X	protein folding/Stress response		DnaJ+DnaJ-X+Dn	DNAJ	DNAJ	DnaJ+DnaJ-X+DnaJ-X	PF14_001	492	DNAJ protein, putative Pf3D7
1E+08	0.42		SIG+DNAJ-X	protein folding/Stress response		SIG+DnaJ-X	SIG	-	DnaJ-X	PF11_002	282	hypothetical protein PF11_0026 Pf3D7
1E+08	0.402		HSP-20-like chaperone(NudC)	protein folding/Stress re	hhpred	-	-	Nimm37	-	PF14_046	333	conserved, unknown function, Pf3D7
3E+08	2.448		HSP70-C-like-IG+TM	protein folding/Stress re	1ud0_A	TM	TM	-	-	PF10_041	182	conserved, unknown function, Pf3D7
3E+08	0.431		SIG+Peptidase_M16+Peptidase_M16_C+Pe	Protein processing	LuxS/MPP-lik	SIG+Peptidase_N	SIG	-	Peptidase_M16+Pep	PF14_038	1560	Stromal-processing peptidase, putative Pf3D7
1E+08	6.285		SIG+SERAprotease	Protein processing		SIG+Pneumo_att	SIG+SERAprotea	GT+SERAprotea	Pneumo_att_G+Pep	PFB0350c	930	serine repeat antigen 3 (SERA-3) Pf3D7
3E+08	4.672		Peptidase_M24/Creatinase/aminopeptidas	Protein processing		Peptidase_M24		-	Peptidase_M24	PFE1360c	327	methionine aminopeptidase, putative Pf3D7
1E+08	-	2.428	Peptidase_C54+Peptidase_C54 (Cysteine Pr	Protein processing/Autophagy		Peptidase_C54+S	-	-	Peptidase_C54+Secr	PF14_017	1124	conserved, unknown function, Pf3D7
3E+08	4.48		TM+TM+Rhomboid+TM	Protein processing; Surface/exported		TM+TM+Rhombo	TM+RHOMBOID	RHOMBOID	Rhomboid	MAL8P1.1	267	rhomboid protease ROM3, putative Pf3D7
1E+08	-	5.327	X-Nterm+TM+TM+RAP(VSR-Rease) relatec	RNA		Atrophin-1+TM+	TM+TM	-	Atrophin-1+RAP	PFE1295c	806	RAP protein, putative Pf3D7
1E+08	-	2.237	RAP(VSR-Rease)	RNA		RAP	RIFIN	-	RAP	PF14_067	532	RAP protein, putative Pf3D7
1E+08	10.51	4.228	Conserved Cs+RAP(VSR-Rease)	RNA		RAP	MED7	-	RAP	PF14_050	1725	RAP protein, putative Pf3D7
3E+08	-	4.46	CCCH(zf-CDGSH)+CCCH(zf-CDGSH-degraded	RNA		zf-CDGSH+TM	TM	-	zf-CDGSH	PF10_022	118	PfMNL-3 CISD1-like iron-sulfur protein, Pf3D7
1E+08	-	4.075	RRM+RRM	RNA		RRM_1	RRM	RRM	RRM_1	PF10_019	322	NOP12-like protein Pf3D7
1E+08	14.22	4.465	RRM	RNA		RRM_1	RRM	RRM	RRM_1	PF10_002	248	RNA binding protein, putative Pf3D7
9E+07	3.368	3.721	RRM+RRM	RNA		RRM_1+RRM_6+	RRM+RRM	RRM+RRM+MIF	RRM_1+RRM_6+Tra	PFF0300w	582	RNA binding protein, putative Pf3D7
1E+08	2.24		CCCH+CCCH+CCCH	RNA		-	ZNKNUCK+PLC	ZNKNUCK+ZNKI	-	PF13_031	1005	conserved, unknown function, Pf3D7
9E+07	0.458		RNA Helicase (3rc3A ATP-dependent RNA h	RNA		Innate_immun+H	IES1	-	Innate_immun+Heli	PFF1140c	1137	ATP dependent DEAD-box helicase, Pf3D7
1E+08	0.391		RRM?+LittleFinger+RRM+C2H2	RNA		zf-RanBP+DUF24	LittleFinger+RRN	MIP-T3+LittleFi	zf-RanBP+DUF2457	PF13_027	1164	ran-binding protein, putative Pf3D7
1E+08	0.455		NUDIX	RNA		NUDIX_2		-	NUDIX_2	PFA_0450	232	mRNA cleavage factor-like protein, Pf3D7
1E+08	-	5.911	RNA-Helicase	RNA metabolism		DEAD+Helicase_(	RNA-Helicase	RNA-Helicase	DEAD+Helicase_C	PF13_003	630	DEAD box helicase, putative Pf3D7
1E+08	12.43		NIC+MA3(TPR)	RNA metabolism		Macoilin+MIF4G+	NIC	MIP-T3+AHD3+	Macoilin+MIF4G+DU	PFL1855w	967	cell cycle control protein, putative Pf3D7
3E+08	6.246		PIN+OB+OB+RNASEII+S1-OB (exonuclease	RNA metabolism	4ifd_j	RNB	RNASEII	SFII-RAD3+RNA	RNB	MAL13P1.	1073	mitotic control protein dis3 homologue, Pf3D7
3E+08	2.344	7.32	SIG+TM+Nop5(3id6)	RNA metabolism	Plasmodium	ETRAMP	SIG+TM	-	ETRAMP	PF14_072	177	early transcribed membrane protein 14.2, Pf
3E+08	0.466		SIG+KOW(hhpred)+Fast_1??+ARL2_Bind_B	RNA metabolism	all hhpred	SIG	SIG	-	-	PFI1468c	1150	leucine-rich repeat protein 8, LRR8 Pf3D7
3E+08	0.452		DUF2418??+Sm (both hhpred)	RNA metabolism	Plasmodium	-	-	-	-	PFI1035w	218	conserved, unknown function, Pf3D7
1E+08	0.363		RnasePH	RNA metabolism		-	RnasePH	RnasePH	-	PFD0605c	332	conserved, unknown function, Pf3D7
3E+08	0.327		NIC	RNA metabolism		Utp14	NIC	NIC	Utp14	PF14_011	947	conserved, unknown function, Pf3D7
1E+08	6.665		Alba	RNA metabolism;chromatin		Alba	Alba	Alba	Alba	PF10_006	107	DNA/RNA-binding protein, putative Pf3D7
1E+08	-	4.083	CCR4	RNA; chromatin		SIG+SMC_N	SIG+IPP	CCR4	SMC_N	PFC0850c	906	endonuclease/exonuclease/phosphatase
1E+08	3.229		RING+RRM+CCCH(inserted?)	RNA;Ubiquitin		zf-RING_4+TT_OI	RING+RRM	DHH+RING+RRI	zf-RING_4+TT_ORF1	PFL1705w	1662	RNA binding protein, putative Pf3D7
1E+08	0.464		MAL7P1.125-repeats aln (hhpred and PSIbla	see aln	see aln	-	-	-	-	MAL7P1.1	880	conserved, unknown function, Pf3D7
1E+08	-	2.905	Adenylate kinase	Signaling		ADK	-	-	ADK	PF10_008	242	adenylate kinase Pf3D7
1E+08	-	2.434	STYKIN	Signaling		Pkinase	DOC	STYKIN+SPX	Pkinase	PFL2280w	909	cyclin g-associated kinase, putative Pf3D7
3E+08	-	2.399	SIG+STYKIN	Signaling		SIG+Pkinase	SIG+STYKIN	STYKIN	Pkinase	PFI0105c	633	Serine/Threonine protein kinase, FIKK Pf3D7
3E+08	-	2.378	TM+phosphodiesterase+TM+TM+TM+TM+	Signaling	alkaline - pho	TM+TM+TM+Acy	TM+TM+TM+TM	MADS+CXXCp+	Acyl_transf_3	PFL0685w	1373	Phosphatidylinositol-glycan biosynthesis
1E+08	-	2.313	IPK	Signaling		IPK	-	-	IPK	PFE0740c	903	kinase, putative Pf3D7
1E+08	15.11		sGTPase+TM?	Signaling		MMR_HSR1+TM	TM	-	MMR_HSR1	PF14_056	797	conserved, unknown function, Pf3D7
1E+08	7.141		C2+Lowcomp	Signaling	Apicomplexa	-	MED3	Asp-B-Hydro_N	-	PF13_017	1001	conserved, unknown function, Pf3D7
3E+08	5.776		STYKIN	Signaling		EDR1+Pkinase	TRANSGLUTAMI	SbcC+CDC27+TI	EDR1+Pkinase	PFI1285w	1179	protein kinase, putative Pf3D7
3E+08	5.124		STYKIN+TM+TM+BZIP+TM+x+TM+TBC+TM-	Signaling		Pkinase+TM+TM-	STYKIN+TM+TM-	STYKIN+TRANS	Pkinase+RabGAP-TB	MAL7P1.1	1936	conserved, unknown function, Pf3D7
9E+07	2.043		sGTP	Signaling		Ras	sGTP+sGTP-Mgl	sGTP-MglA	Ras	PFF0810c	516	conserved, unknown function, Pf3D7
3E+08	0.441		EF-hand	Signaling		-	-	-	-	PFA_0305	215	conserved, unknown function, Pf3D7
9E+07	0.44		EGF	Signaling		EF-hand_7	EFHAND	EFHAND	EF-hand_7	PFF0265c	160	calcium-binding protein, putative Pf3D7
1E+08	0.329		14-3-3	Signaling		14-3-3	-	-	14-3-3	PF14_022	425	conserved, unknown function, Pf3D7
3E+08	3.705		STYKIN+Beta-lactamase+C-rich	Signaling	C-term 2/201	ABC1+Beta-lacta	STYKIN	STYKIN+Asp-B-H	ABC1+Beta-lactamas	PF14_014	2763	Atypical protein kinase, ABC-1 family, Pf3D7
1E+08	3.204		TM+fragment of a kinase	Signaling	Apicomplexa	TM	TM	-	-	PF14_073	347	Serine/Threonine protein kinase, FIKK Pf3D7
3E+08	-	6.107	SIG	Surface/exported protei	Falciparum o	SIG	SIG	-	-	PFE1615c	161	Plasmodium exported protein, Pf3D7
1E+08	-	5.409	SIG+PF07_0087-C..CxxC….HxRxG...C (PF07_	Surface/exported protei	Apicomplexa	SIG	SIG	-	-	PF07_008	244	conserved, unknown function, Pf3D7
1E+08	-	5.316	SIG+ACBP+ANK	Surface/exported protein		SIG+ACBP+Ank_2	SIG+ANK	ANK	ACBP+Ank_2	PF11_019	371	conserved Pf3D7
1E+08	-	5.14	TM+TM+DHHC-ZNF+TM+TM	Surface/exported protein		TM+zf-DHHC	TM+TM+DHHC-Z	DHHC-ZNF	zf-DHHC	MAL13P1.	313	DHHC-type zinc finger protein, putative Pf3D7
1E+08	-	4.609	common+TM+TM+TM+TM+TM	Surface/exported protei	Apicomplexa	TM+TM+TM+TM	TM+TM+TM+TM	-	-	PFL0775w	293	conserved, unknown function, Pf3D7
3E+08	-	3.653	TM	Surface/exported protei	Apicomplexa	TM	TM	-	-	PFL1432w	82	conserved Pf3D7
1E+08	-	3.366	TM+TM+X+TM+TM+TM+TM	Surface/exported protei	Plasmodium	TM+TM+TM+TM	TM+TM+TM+TM	-	-	PF14_059	523	conserved, unknown function, Pf3D7
1E+08	-	2.646	SIG+Rifin_STEVOR	Surface/exported protein		Rifin_STEVOR	RIFIN	RIFIN	Rifin_STEVOR	PFL2645c	317	rifin Pf3D7
1E+08	-	2.375	HEAT repeats+TM+TPR(Vac14_Fab1_bd)	Surface/exported protein		DUF2076+Vac14_	TM	UBC+SPX+8TMI	DUF2076+Vac14_Fal	PFL1240c	1501	conserved Pf3D7
1E+08	-	2.327	SIG+Duffy_binding+Duffy_binding+EBA-175	Surface/exported protein		SIG+Duffy_bindir	SIG+VAR+TM	MIP-T3	Duffy_binding+Duffy	PFA_0125	1567	erythrocyte binding antigen-181 Pf3D7
1E+08	-	2.26	SIG+Rifin_STEVOR	Surface/exported protein		SIG+Rifin_STEVO	RIFIN	RIFIN	Rifin_STEVOR	PF14_000	339	rifin Pf3D7
3E+08	-	2.123	Lowcomp+TM+TM+TM+TM+TM+TM+TM	Surface/exported protein		HlyIII	TM+TM+TM+TM	-	HlyIII	PF14_052	282	hemolysin, putative Pf3D7
1E+08	-	2.111	TM	Surface/exported protei	Falciparum o	TM	TM	-	-	PFL2525c	378	Plasmodium exported protein Pf3D7
1E+08	11.95		SIG+TM+TM+TM+Tox-SUFU-associated	Surface/exported protein		SIG+TM+TM+TM	SIG+TM+TM+TM	Tox-SUFU-assoc	-	MAL8P1.5	455	conserved, unknown function, Pf3D7
1E+08	11.12		TM+TM+TM+TM+TM+TM	Surface/exported protei	Apicomplexa	RP1-2+TM+TM+	TM+TM+TM+TM	PUA+EP1	RP1-2+Nucleoplasmi	PF11_043	1828	conserved Pf3D7
1E+08	10.25		SIG+Rifin_STEVOR	Surface/exported protein		SIG+Rifin_STEVO	RIFIN	RIFIN	Rifin_STEVOR	PFI1810w	359	rifin Pf3D7
3E+08	9.851		SIG+TM+TM+EGF+TM	Surface/exported protein		SIG+TM+TM+TM	SIG+TM+TM+EG	EGF+PrsW	-	PFI0900w	1720	conserved, unknown function, Pf3D7
3E+08	8.787		SIG+TM+TM+TM+TM+TM+TM+TM	Surface/exported protein		SIG+PSS+TM	SIG+TM+TM+TM	-	PSS	MAL13P1.	357	phosphatidylserine synthase I, putative Pf3D7
1E+08	6.732		TM+TM+TM+TM+TM	Surface/exported protei	Plasmodium	TM+TM+TM+TM	TM+TM+TM+TM	SbcC	DUF2457+DUF3300	MAL13P1.	5415	conserved, unknown function, Pf3D7
3E+08	5.039		Duffy_binding+PFEMP+PF12879_SICA_C	Surface/exported protei	PF12879 SICA	Duffy_binding+PI	-	MIP-T3+MIP-T3	Duffy_binding+PFEM	PF11_050	6093	Antigen 332, DBL-like protein Pf3D7
1E+08	4.332	4.839	SIG+erythrocyte mem (PfemP)	Surface/exported protein		ATS	SIG	-	ATS	PFB1045w	431	Pf erythrocyte membrane protein 1 truncated
3E+08	3.427	5.811	SIG+Rifin_STEVOR	Surface/exported protein		SIG+TM	RIFIN+TM	RIFIN	-	PF14_075	358	conserved, unknown function, pseudogene
1E+08	3.1	0.396	SIG+Rifin_STEVOR	Surface/exported protein		SIG+Rifin_STEVO	RIFIN	RIFIN	Rifin_STEVOR	PFD0040c	329	rifin Pf3D7

1E+08	3.071		SIG+Tox-ALFMPTase(Zincin-Metalloproteas	Surface/exported protein		SIG	SIG	Tox-ALFMPTase	-	PFD0425w	984	conserved, unknown function, Pf3D7
1E+08	3.06		TM+TM+TM+TM+LowComp+TM+TM	Surface/exported protei	Falciparum o	TM+TM+TM+TM	TM+TM+TM+TM	FilH	Secretin_N_2	MAL7P1.1	779	conserved, unknown function, Pf3D7
3E+08	2.94		SIG+Rifin_STEVOR	Surface/exported protein		SIG+Rifin_STEVO	RIFIN	RIFIN	Rifin_STEVOR	PFB1035w	330	rifin Pf3D7
1E+08	2.841		SIG+TM+(conserved WDY)	Surface/exported protei	Plasmodium	SIG+TM	SIG+TM	-	-	PFB0930w	225	Plasmodium exported protein (hyp9) Pf3D7
3E+08	2.72		SIG+Methylase	Surface/exported protein		SIG+Cons_hypoth	SIG+DNAMETHY	SPX+METHYLAS	Cons_hypoth95	MAL13P1.	561	N6-adenine-specific methylase, putative Pf3D7
1E+08	2.643		SIG+Rifin_STEVOR	Surface/exported protein		SIG+Rifin_STEVO	RIFIN	RIFIN	Rifin_STEVOR	PF11_0516	308	stevor, putative [Plasmodium falciparum 3D7]
3E+08	2.631		SIG+Rifin_STEVOR	Surface/exported protein		SIG+Rifin_STEVO	RIFIN+TM	RIFIN	Rifin_STEVOR	PFB0035c	375	rifin Pf3D7
1E+08	2.509		SIG+PRESAN+PRESAN	Surface/exported protei	Plasmodium	SIG+PRESAN+Nop	SIG	SWC3+MIP-T3	PRESAN+Nop14+PRE	PFL0050c	653	Plasmodium exported protein (PHISTb) Pf3D7
1E+08	2.484		SIG+Rifin_STEVOR	Surface/exported protein		SIG+Rifin_STEVO	RIFIN	RIFIN	Rifin_STEVOR	PF13_0005	331	rifin Pf3D7
3E+08	2.466		SIG+LowComp+TM+TM	Surface/exported protei	Plasmodium	SIG+TM+TM	SIG+TM+TM	7TMR-HD	-	PF11_0014	231	Maurer's Cleft 2 transmembrane domain 11.1
1E+08	2.294		LowComp+TM+TM	Surface/exported protei	Apicomplexa	TM+TM	TM+TM	-	-	MAL7P1.9	179	conserved, unknown function, Pf3D7
1E+08	2.124		SIG+TM+TM	Surface/exported protei	Plasmodium	SIG+TM+TM	SIG+TM+TM	-	-	PFD1205w	230	Plasmodium exported protein (hyp15), Pf3D7
1E+08	2.051		SIG+Rifin_STEVOR	Surface/exported protein		SIG+Rifin_STEVO	RIFIN+TM	RIFIN	Rifin_STEVOR	PF11_0013	259	stevor, putative, degenerate Pf3D7
3E+08	0.464		TM+TM+TM+TM+TM+TM+TM+TM	Surface/exported protein		TM+TM+TM+TM	TM+TM+TM+TM	-	-	PF14_0778	373	conserved, unknown function, Pf3D7
1E+08	0.461		SIG+Rifin_STEVOR	Surface/exported protein		SIG+Rifin_STEVO	RIFIN	RIFIN	Rifin_STEVOR	PFD0645w	346	rifin Pf3D7
3E+08	0.445		SIG+Apicomplexa-specific	Surface/exported protei	Apicomplexa	SIG	SIG	Eukglutathiones	-	PF11_0069	266	conserved, unknown function, Pf3D7
1E+08	0.436		SIG+(conserved DxxD…. HDIN)	Surface/exported protei	Plasmodium	SIG	SIG	-	-	PFC0795w	202	conserved, unknown function, Pf3D7
1E+08	0.418		TM+LowComp	Surface/exported protei	Plasmodium	TM	TM	-	-	PFI1055w	421	conserved, unknown function, Pf3D7
1E+08	0.382		TM+TM+TM+TM	Surface/exported protei	Plasmodium	TM+TM+TM+TM	TM+TM+TM+TM	SbcC	-	PFE1180c	1482	conserved, unknown function, Pf3D7
1E+08	0.37		LowComp+TM+TM+TM+TM	Surface/exported protei	Plasmodium	Rib_recp_KP_reg	TM+TM+TM+TM	MIP-T3+7TMR-I	Rib_recp_KP_reg	MAL13P1.	324	conserved, unknown function, Pf3D7
1E+08	0.333		SIG+Lowcomp	Surface/exported protei	Plasmodium	SIG	SIG	-	-	PF10_0030	553	conserved, Pf3D7
1E+08	0.305		SIG+LowComp	Surface/exported protei	Plasmodium	SIG	SIG+IES4	SPX	-	PF13_0361	897	conserved, unknown function, Pf3D7
3E+08	4.004		SIG+PF07422-s48_45/2ymo	Surface/exported protei	Plasmodium	SIG+Secretin_N_	SIG	-	Secretin_N_2	PFC0750w	969	conserved, unknown function Pf3D7
3E+08	0.412		Adaptin_N+Adaptin_N (TPR repeats)	Trafficking		Adaptin_N+Adap	IBD-HTH	Asp-B-Hydro_N	Adaptin_N+Adaptin_	PFI0200c	1388	conserved, unknown function, Pf3D7
3E+08	2.424		ZnR/zf-Tim10_DDP	Trafficking (mitochondrial)		zf-Tim10_DDP	-	-	zf-Tim10_DDP	PFL2065c	95	mitochondrial inner membrane translocase
1E+08	0.467		ZnR/zf-Tim10_DDP	Trafficking (mitochondrial)		zf-Tim10_DDP	-	-	zf-Tim10_DDP	PFE0140c	82	zinc binding protein, putative Pf3D7
1E+08	-	6.182	SIG+Lowcomp+GOLD+TM	Trafficking (vesicular)		SIG+EMP24_GP2	SIG+GOLD+TM	GOLD	EMP24_GP25L	PFD1037w	385	conserved, unknown function, Pf3D7
1E+08	3.18		Sig+TM+TM(Got1)	Trafficking (Vesicular)		Got1	SIG+TM	-	Got1	PFD0930w	136	CGI-141 protein homolog, putative Pf3D7
3E+08	0.264		DUF1712/Mon1/CCZ1	Trafficking (Vesicular)	related to (SI	DUF1712	-	-	DUF1712	PF11_0084	713	conserved Pf3D7
3E+08	3.792		Rab7 sGTP	Trafficking(Vesicular)		Ras	sGTP	sGTP	Ras	PFI0155c	206	PfRab7, GTPase Pf3D7
1E+08	4.271		RPB10-HTH	Transcription		RNA_pol_N	RPB10-HTH	RPB10-HTH	RNA_pol_N	PF07_0027	69	DNA-directed RNA polymerase, Pf3D7
3E+08	-	2.187	(wHTH+C-rich)/RNA_pol_Rpc34+TM	Transcription		RNA_pol_Rpc34+	TM	SbcC	RNA_pol_Rpc34	PF14_0207	311	RNA polymerase subunit, putative Pf3D7
1E+08	2.643		HTH (Apicomplexa 3 iteration)	Transcription		HTH_3	CHTH	CHTH	HTH_3	PFC0506w	136	conserved, unknown function, Pf3D7
1E+08	-	3.39	Sin_N+HTH?+Sin_N (2/2015 Job-ID: 187667	Transcription	Sin_N - DNA-	Sin_N+TT_ORF1+	-	-	Sin_N+TT_ORF1+Sin_	PF14_0561	865	conserved, unknown function, Pf3D7
1E+08	-	4.251	SIG+Eif-1 (S1)	Translation	(S1 - B>Plasm	SIG+eIF-1a	SIG	S1	eIF-1a	PF14_0658	182	translation initiation factor EF-1, putative Pf3D7
1E+08	-	2.765	TM+TM+PINT-HTH	Translation		TM+TM+PCI	TM+TM+PINT	PINT	PCI	PFE1405c	517	eukaryotic translation initiation factor 3
1E+08	-	2.516	TetQ GTPase	Translation		GTP_EFTU+Yae1_	sGTP	-	GTP_EFTU+Yae1_N+	PFL1710c	1161	tetQ family GTPase, putative Pf3D7
1E+08	23.31		Ribosomal S11 (OB-S1?)	Translation		Ribosomal_S17	-	-	Ribosomal_S17	PFC0775w	161	40S ribosomal protein S11, putative Pf3D7
1E+08	21.36	6.695	GTP_EFTU+GTP_EFTU_D2+GTP_EFTU_D3	Translation		GTP_EFTU+GTP_	sGTP-MglA	SbcC	GTP_EFTU+GTP_EFT	PF11_0245	555	translation elongation factor EF-1, Pf3D7
9E+07	14.34		sGTP (IF2)	Translation		SMC_N+GTP_EFT	SIG+sGTP	MIP-T3+sGTP	SMC_N+GTP_EFTU+	PFF0345w	977	translation initiation factor IF-2, putative Pf3D7
1E+08	13.15	4.087	Ribosomal_L44	Translation		Ribosomal_L44		-	Ribosomal_L44	PFC0200w	104	60S Ribosomal protein L44, putative Pf3D7
1E+08	6.815		Ribosomal_L24e	Translation		Ribosomal_L24e	-	-	Ribosomal_L24e	PF13_0049	162	60S ribosomal protein L24, putative Pf3D7
3E+08	4.817	3.104	trnA_Synthetase+DALR-anticodon-binding	Translation		SIG+tRNA-synt_1	SIG	-	tRNA-synt_1e	PF10_0149	677	cysteinyl-tRNA synthetase, putative Pf3D7
1E+08	4.759		tRNA_anti-codon-OB-fold+wHTH(hhpred)	Translation		tRNA_anti-codon	-	-	tRNA_anti-codon	PF11_0332	273	conserved Pf3D7
1E+08	4.616		PCRF+RF-1	Translation		PCRF+RF-1	-	SbcC	PCRF+RF-1	MAL7P1.2	352	peptide chain release factor, putative Pf3D7
1E+08	0.354		Ribosomal_L28	Translation		Ribosomal_L28	-	-	Ribosomal_L28	PF14_0539	281	mitochondrial ribosomal protein L28, Pf3D7
1E+08	8.01		SAM-Methylase	translation; RNA		Methyltransf_8	SAM-methylase	SAM-methylase	Methyltransf_8	PFI1235w	413	methyltransferase, putative Pf3D7
1E+08	-	3.554	V_ATPase_I+V_ATPase_I+TM+TM+TM+TM	Transport		V_ATPase_I+PBP	TM+TM+TM+TM	-	V_ATPase_I+PBP1_T	PF08_0113	1053	vacuolar proton translocating ATPase subunit A
1E+08	11.43	5.254	TM+CDC50+TM	Transport		TM+CDC50	TM+TM	-	CDC50	PF07_0078	462	transmembrane protein, putative Pf3D7
1E+08	7.554		TM+TM+TM+TM+TM+ABC-ATPase+TM+TM	Transport		TM+TM+TM+TM	TM+TM+TM+TM	sigma+PTSEIIC+	ABC_tran+Herpes_L	PFL1410c	2108	ABC transporter, (CT family) Pf3D7
3E+08	6.016		TM+TM (CorA)	Transport		CorA	TM+TM	-	CorA	MAL13P1.	468	unnamed protein product Pf3D7
3E+08	0.393		TM+TM+TM+TM+Mito_carr+TM	Transport		Mito_carr+Mito_	TM+TM+TM+TM	-	Mito_carr+Mito_car	PFA_0415	330	mitochondrial carrier protein, putative Pf3D7
3E+08	4.453		Ulp1. Cysteine Protease/Ulp1 protease/Pep	Ubiquitin		Peptidase_C48	Ulp1	SbcC+Ulp1	Peptidase_C48	PFL1635w	1026	Ulp1 protease, putative Pf3D7
1E+08	-	0.443	SIG+UBA+EF-Ts-domain	Ubiquitin	hhpred	SIG	SIG	-	-	PF14_0406	402	conserved, unknown function, Pf3D7
3E+08	14.79		SIG+UFD(Double-psi-beta-barrel)	Ubiquitin		SIG+UFD1	SIG+UFD	-	UFD1	PFI0810c	296	apicoplast Ufd1 precursor Pf3D7
3E+08	3.958		Cysteine Protease/UBP12/Peptidase_C12	Ubiquitin		Peptidase_C12	UBP12	UBP12	Peptidase_C12	PF14_0576	232	ubiquitin carboxyl-terminal hydrolase, Pf3D7
3E+08	2.943		SIG+RING	Ubiquitin		SPX+zf-C3HC4_4	SIG+RING	SPX+RING	SPX+zf-C3HC4_4	PFB0440c	568	conserved Pf3D7
3E+08	0.429		SIG+ubiquitin(betagrasp)+alpha-helical+TM	Ubiquitin		SIG+ubiquitin+TM	SIG+TM+TM+TM	UBI	ubiquitin	PF08_0067	373	conserved, unknown function, Pf3D7
1E+08	0.323		UBHYD(with insert)	Ubiquitin		SART-1+UCH+UC	UBHYD	UBHYD	SART-1+UCH+UCH	PF14_0145	1384	ubiquitin C-terminal hydrolase, putative Pf3D7
9E+07	0.423		Cullin+Cullin+Cullin	Ubiquitin		Cullin+Cullin+Cull	CULLIN	CULLIN	Cullin+Cullin+Cullin	PFF1445c	1129	cullin-like protein, putatiave Pf3D7
3E+08	-	3.701	Baculo_Helicase/PF04735??(hhpred only)	Plasmodium only	Plasmodium	-	-	ISOFLAVOMETH	-	MAL13P1.	2183	conserved, unknown function, Pf3D7
1E+08	-	2.416	HEAT repeats			HEAT_2+HEAT_2	ImmHEAT	-	HEAT_2+HEAT_2	PF13_0013	411	PBS lyase HEAT-like protein, putative Pf3D7
1E+08	18.98		(camp-regulatory-sbubunit?-4f9k)		Plasmodium only.web-hhpred-2 helix hit			-	-	PF10_0176	282	conserved Pf3D7
1E+08	8.617	11.13	HEAT Rpt(200..416)			-	-	-	-	PFE1060c	1526	conserved, unknown function, Pf3D7
1E+08	4.993		(HTH?)+ZnR			-	-	-	-	PFC0600w	249	conserved, unknown function, Pf3D7
1E+08	3.696		GAS (coiled-coil)			GAS		-	GAS	PFL2040w	450	conserved, unknown function, Pf3D7
1E+08	0.491		Thioredoxin					-	-	PFI0245c	281	conserved, unknown function, Pf3D7
